# From Fibrils to Toughness: Multi-Scale Mechanics of Fibrillating Interfaces in Stretchable Electronics

**DOI:** 10.3390/ma11020231

**Published:** 2018-02-02

**Authors:** Olaf van der Sluis, Tijmen Vermeij, Jan Neggers, Bart Vossen, Marc van Maris, Jan Vanfleteren, Marc Geers, Johan Hoefnagels

**Affiliations:** 1 Department of Mechanical Engineering, Eindhoven University of Technology, P.O. Box 513, 5600 MB Eindhoven, The Netherlands; t.vermeij@student.tue.nl (T.V.); jan.neggers@lmt.ens-cachan.fr (J.N.); b.vossen@gmail.com (B.V.); m.v.maris@tue.nl (M.v.M.); m.g.d.geers@tue.nl (M.G.); j.p.m.hoefnagels@tue.nl (J.H.); 2 Philips Research Laboratories, High Tech Campus 4, 5656 AE Eindhoven, The Netherlands; 3 Center for Microsystems Technology (CMST), Ghent University—IMEC, Technologiepark 15, B-9052 Gent-Zwijnaarde, Belgium; Jan.vanfleteren@ugent.be

**Keywords:** stretchable electronics, interface delamination, cohesive zone, fibrillation, multi-scale analysis, PDMS, traction-separation law, peel test, fracture process zone

## Abstract

Metal-elastomer interfacial systems, often encountered in stretchable electronics, demonstrate remarkably high interface fracture toughness values. Evidently, a large gap exists between the rather small adhesion energy levels at the microscopic scale (‘intrinsic adhesion’) and the large measured macroscopic work-of-separation. This energy gap is closed here by unravelling the underlying dissipative mechanisms through a systematic numerical/experimental multi-scale approach. This self-containing contribution collects and reviews previously published results and addresses the remaining open questions by providing new and independent results obtained from an alternative experimental set-up. In particular, the experimental studies on Cu-PDMS (Poly(dimethylsiloxane)) samples conclusively reveal the essential role of fibrillation mechanisms at the micro-meter scale during the metal-elastomer delamination process. The micro-scale numerical analyses on single and multiple fibrils show that the dynamic release of the stored elastic energy by multiple fibril fracture, including the interaction with the adjacent deforming bulk PDMS and its highly nonlinear behaviour, provide a mechanistic understanding of the high work-of-separation. An experimentally validated quantitative relation between the macroscopic work-of-separation and peel front height is established from the simulation results. Finally, it is shown that a micro-mechanically motivated shape of the traction-separation law in cohesive zone models is essential to describe the delamination process in fibrillating metal-elastomer systems in a physically meaningful way.

## 1. Introduction

Close integration of micro-electronic devices with biological tissue would facilitate a realm of ground-breaking (in-body) applications: from surgical and diagnostic implements that naturally integrate with the human body, such as eye-implanted retina-shaped photo-sensor arrays, electrode array probes for deep brain, heart, or nerve stimulation, chips and sensors on the tip of minimally invasive instruments, and cardiac diagnostics, to numerous ex-vivo applications, such as sensory skin for robotics and prostheses and wearable health monitoring e.g., [1,2,3,4,5,6,7,8,9]. The main challenge of such devices is to match their shape and mechanical properties such as flexibility, stretchability and texture as closely as possible to that of the tissue. However, biological systems are soft, deformable, and curved, whereas conventional electronic devices are stiff, brittle, and flat. In Harris et al. [10], besides discussing the different classes of flexible electronic devices, the importance of understanding the mechanics and critical failure mechanisms to ensure proper reliability and lifetime of any flexible device is emphasized. Yu et al. [11] present a review that focuses on suitable materials, processing techniques and device applications, and conclude that “stretchable electronics bring electronics closer to humans”. Klein et al. [12] propose qualification test methods specifically designed for stretchable electronics, which result in several failure modes that closely resemble the actual device.

It is not the aim of this paper to present a complete overview of all developments in this continuously evolving research field. Instead, the focus is on the specific challenge to accommodate the large difference in stiffness values between the copper conductors and the rubber encapsulant specifically at large stretchability. Ensuring reliability of the electronic circuits is a challenge due to two critical failure mechanisms: cohesive (material) and adhesive (interface) fracture. Ductile (cohesive) fracture of the metal interconnects directly leads to device failure due to the loss of its electrical functionality. To prevent this failure mechanism, the metal conductors are serpentine shaped [13,14,15], and subsequently accommodate large deformation of the substrate while small strains are present in the metal. Recently, an ultra-compliant freestanding interconnect structure was reported that exhibited an impressive elastic stretch beyond 2000%, while manufactured in a CMOS (Complementary Metal Oxide Semiconductor)-compatible process [16]. Adhesive fracture between the copper conductors and the stretchable substrate, which is the focus of this work, may result in electrical shorts and ductile fracture of the conductor due to necking, which in turn leads to electrical failure of the stretchable device [17,18,19]. In large elastic-mismatch elastomer-metal interfaces, which are also present in the more recently introduced metallic nanostructures-elastomer systems [20,21,22,23], adhesive failure typically occurs through stretching-induced fibrillation [24,25] which can result in remarkably high values of the macroscopic interface fracture toughness $\Gamma_{i}$. Values of $\Gamma_{i}>1000$ J$m^{-2}$ have been reported for Cu-PDMS (Poly(dimethylsiloxane)) systems [25,26,27,28], which is significantly larger than the typical values of the adhesion energy of metal–rubber valence bonds, $0.1<\Gamma_{0}<10$ J$m^{-2}$ [29,30].

In [31,32], fibrillar structures are studied that are in direct contact with the substrate, resulting in the loss of elastically stored energy in the fibril upon detachment, which is considered to be the main dissipative mechanism in these systems. The contact and adhesion between a PDMS fibrillar structure and a rigid substrate are studied in [33,34]. The adhesion enhancement has been studied in more detail in e.g., [35,36,37]. In [38], finite strain elasticity is used to determine the energy contribution of the fibrils. The aforementioned studies do not account for the energy storage and loss in the bulk layer interconnecting the fibrillar structure. More recently, Zhao and co-workers [39,40] reported very high work-of-separation (WOS) values of intrinsically tough hydrogels, which was achieved by chemically anchoring the hydrogel on a solid substrate. The anchoring mechanism resulted in large deformations in the hydrogel in which a significant amount of energy was dissipated by reversible crosslinking and chain scission and contributed to the interfacial toughness. Fibrillar hierarchical interfaces are also encountered in nature, in order to facilitate temporary adhesion to arbitrary structures [41].

Fibrillation micro-mechanics have been studied intensively, especially for viscoelastic pressure-sensitive adhesives (PSAs) [42]. Creton and co-workers [43,44,45] used tack tests to better understand which parameters control the fibrillation process. However, there are two main differences between PSAs and our material system: (i) PSA fibrils are viscoelastic while PDMS is hyperelastic; and (ii) PSA fibril lengths are in the millimeter range while the PDMS fibrils are only tens of micrometers long. As a result, the relative contribution of the fibril elongation to the WOS for both systems appears to be fundamentally different: for PSAs, the largely elongated viscoelastic fibrils contribute significantly to the energy dissipation during fibrillation, while, for PDMS, fibril elongation constitutes only a minor contribution to the WOS value.

Adhesion is often measured at the macroscopic scale, e.g., [46,47]. Due to the macroscopic approach, quantitative comparison of adhesion results obtained from different test methods is not trivial as different dissipative mechanisms across the scales can contribute differently to the macroscopic adhesion energy. An intriguing example of this difference in micro-scale dissipation mechanisms is found in the measured WOS values in a polymer-coated metal, of which values of 2 J$m^{-2}$ [48], 30 J$m^{-2}$ [49] and 194 J$m^{-2}$ [50] have been reported by different techniques on exactly the same material system. What is actually measured is the practical work of adhesion, or WOS [46,50]:

(1) $$\Gamma_{i}=\Gamma_{0}+\Gamma_{D}$$

where $\Gamma_{0}$ is the thermodynamic work of adhesion (or, similarly, the earlier mentioned adhesion energy of metal-rubber valence bonds), and $\Gamma_{D}$ contains all dissipative terms that contribute to the WOS value, such as plastic dissipation and friction. It can thus be argued that the macroscopic adhesion properties are a complex function of all dissipative mechanisms across the scales. Thorough understanding of the significance of each of these dissipative mechanisms is essential in order to establish physically correct, unambiguous, values of the adhesion properties, which can only be achieved by proper multi-scale techniques.

**Figure 1:**
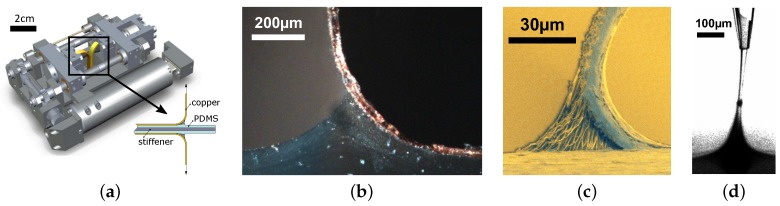
Illustration of the multi-scale character of fibrillating metal-elastomer interfaces: (**a**) the test specimen at macro-scale; (**b**) the PDMS (Poly(dimethylsiloxane)) lift-off geometry (FPZ) at meso-scale; (**c**) the fibrils at meso-scale; (**d**) a fibril at micro-scale.

This contribution therefore aims to unravel the huge gap that exists between the microscopic adhesion energy and the macroscopic WOS of the aforementioned Cu-PDMS systems by means of a multi-scale experimental and numerical analysis. To this end, the key results from a range of previously-reported, experimental and numerical studies are collected and reviewed, while addressing the remaining open questions through enriching the analysis by newly designed, fully independent, micro-tests to come to a consistent, self-containing, overall description of the multi-scale fibrillation process. The structure of this paper follows the multi-scale character of fibrillating Cu-PDMS interfaces, by zooming in from the macroscopic to the microscopic scale, as illustrated in Figure 1. First, 0${}^{\circ }$ and 90${}^{\circ }$ peel test experiments will be discussed, where samples with high and low copper surface roughness are characterized from their resulting force-displacement curves. Next, a finite element (FE) model of the mixed-mode peel test experiments employing large displacement cohesive zone (CZ) elements is presented. CZ parameters (strength, toughness, and mode mixity) are extracted for both roughnesses. At the micron scale, in situ high magnification Environmental Scanning Electron Microscopy (ESEM) and Confocal Microscopy are applied to visualize the fibrillation at the peel front while quantitatively matching the 3D topologies of the delaminated fracture surfaces, and the PDMS residue on the copper surface. An alternative peel test that allows for visualization from the side face is introduced to identify the actual initiation mechanism of PDMS fibrillation. Subsequently, results of single and multiple fibril FE models that describe instantaneous fibril failure are used to identify the relevant dissipative mechanisms. A quantitative relation between the height of the PDMS lift-off geometry (i.e., the fracture process zone, FPZ) and the calculated WOS is established and validated against experimental results. Finally, it is shown that a micro-mechanically motivated shape of the traction-separation law used in the CZ model is essential to describe the delamination process in fibrillating metal-elastomer systems in a physically meaningful way.

## 2. The Peel Test Experiments

To characterize the mixed-mode adhesion properties, 0${}^{\circ }$ and 90${}^{\circ }$ peel tests are performed on copper film on PDMS substrate samples. To quantify the effect of surface roughness on the resulting dissipative mechanisms during peeling, copper films with two different surface roughness values (‘smooth’ and ‘rough’) are applied. The peel test samples, with in-plane dimensions 50 × 7 mm${}^{2}$, are cut from bi-layer sheets consisting of 17 μm copper foil on top of a PDMS sheet of either 1.0 mm or 0.75 mm thickness. The used PDMS is Sylgard 186 (Dow Corning, Midland, MI, USA), while the copper foil is TW-YE rolled foil from ArcelorMittal (London, UK) with one untreated side (roughness $R_{a}\approx 0.5\;\mu$m), while the opposite side is treated with an electroplating step to increase the roughness ($R_{a}\approx 1.9\;\mu$m). The bi-layer sheets are made by moulding the PDMS to either side of the copper foil, after which the PDMS is fully cured.

To study the mixed-mode behaviour of the Cu-PDMS interface, two peel experiments are applied with distinct macroscopic opening angles. To facilitate proper imaging of the peel front, the samples are designed such that two stationary peel fronts occur during delamination. For this purpose, two bi-layer samples are glued to a 0.5 mm thick aluminium stiffener for the 90${}^{\circ }$ peel experiment (see the inset in Figure 1a). The 0${}^{\circ }$ peel experiments require a pre-crack in the foil, which is initiated by pre-damaging the copper with a knife-edge along a line perpendicular to the loading direction. The copper will fracture upon loading, thereby transferring the load to the PDMS. Due to the large elastic mismatch, the interface is loaded in shear. The peel tests are performed with a micro tensile stage (Kammrath and Weiss GmbH, Dortmund, Germany) using a 20 N loadcell [28]. The small size of the tensile stage allows operation beneath the objective of an optical microscope, or inside the vacuum chamber of an ESEM. The resulting force-displacement curves of both tests show the two typical peel regimes: (i) the initiation part (increasing force with increasing displacement), and (ii) the steady-state part (an approximately constant force with increasing displacement) [28]. When neglecting dissipation due to film extension, the following relation between steady-state peel force and interface toughness holds

(2) $$\Gamma_{i}=\frac{P_{f}}{w}\left(1-\cos\theta \right)$$

where $P_{f}$ is the plateau force (also referred to as ‘peel force’), *w* the width of the sample (7 mm in these experiments), and θ the peel angle [51,52,53]. More details on the actual peel experiments described above are provided in [28].

## 3. The Peel Test Simulation Model

To characterize the adhesive properties, FE models of both peel test configurations are used. Here, the PDMS is described by a hyper-elastic neo-Hookean model, defined by the strain energy $W=C_{\mathrm{nH}}\left(\lambda_{1}^{2}+\lambda_{2}^{2}+\lambda_{3}^{2}-3\right)$, with $\lambda_{i}$ the principal stretches and $C_{\mathrm{nH}}=0.5\;G$ with *G* the rubber elastic (shear) modulus. From uniaxial and planar extension experiments, $C_{\mathrm{nH}}=0.165$ MPa is identified. The copper film is modelled as an elasto-plastic material, in which the hardening behaviour (i.e., yield stress as function of equivalent plastic strain) is obtained from uniaxial tensile experiments for logarithmic strains up to 0.1 [26]. The delamination process during peeling is described by a fibril-based mode-dependent interface model with a large displacement formulation employing first Piola–Kirchhoff tractions, as adopted from [54]. The opening and traction vectors are not decomposed with respect to a local basis but resolved globally and, thus, no distinction needs to be made between the normal and tangential directions in the constitutive formulation. A single exponential relation between the traction *t* and separation δ (called the traction-separation law, TSL) is formulated as follows

(3) $$t=\frac{\Gamma_{i}}{\delta_{c}}\frac{\delta }{\delta_{c}}\exp\left(-\frac{\delta }{\delta_{c}}\right)\exp\left(\alpha \frac{d}{2}\right)$$

in which the traction vector t=te and the opening displacement vector δ=δe, where e is a unit vector connecting associated points of the interface (i.e., the fibril) and is resolved in the current configuration, $\Gamma_{i}$ the WOS and $\delta_{c}$ is the separation value at which the maximum traction (interface strength) $t_{\max}$ is reached:

(4) $$\delta_{c}=\frac{\Gamma_{i}}{\exp\left(1\right)t_{\max}}$$

The parameter *d* describes the crack opening mode: *d* varies between 0 (mode I) and 2 (mode II), with intermediate values representing a mixed-mode opening. The parameter α defines the mode-dependency: the WOS is higher (α>0) or lower (α<0) in mode II than in mode I. Irreversibility is taken into account by assuming linear elastic unloading to the origin. More details can be found in [50].

The CZ parameters have been identified by matching the calculated and measured global force-displacement curves and the local PDMS lift-off geometries at the delamination front, i.e., the fracture process zone [26,28]. For the low roughness interface, $\Gamma_{i}=100$ J$m^{-2}$, $t_{\max}=0.50$ MPa, and α=0.1 were identified, while for the high roughness interface, $\Gamma_{i}=300$ J$m^{-2}$, $t_{\max}=0.75$ MPa, and α=0.0 resulted in the best match between simulation and experiment. Therefore, from the macroscopic analysis, it appears that the high roughness interface is tougher and stronger, while both interfaces show a negligible mode angle dependency (reflected by the low values of α).

Neggers et al. [28] validated the proposed model by comparing strain fields in the PDMS material during a 0${}^{\circ }$ peel test. This loading angle resembles the actual in-plane stretching in a real device [24]. The maximum difference in these strain fields was less than 10% from which it was concluded that the model adequately describes the mechanics of the peel test for this particular material system at the macroscopic and mesoscopic scale. Furthermore, in-plane stretching results obtained for a different sample batch with a similar approach, as reported in [26], were compared to experimental results as well. For two different interconnect designs (the horseshoe and the zigzag shape), it could also be concluded that these models accurately describe the delamination mechanism at the macro- and mesoscopic scales.

**Figure 2:**
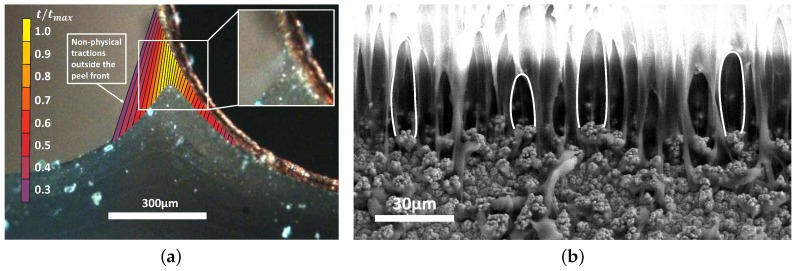
(**a**) illustration of the presence of non-physical tractions in regions where there is no material (left side of the PDMS lift-off geometry) caused by the macroscopic CZ model; (**b**) the copper surface roughness causes PDMS interlocking in the roughness valleys and initiation of fibrillation at the roughness peaks.

However, Hoefnagels et al. [25] observed a large mismatch between the critical opening of the macroscopic CZ model and the fibril length at failure, which was attributed to the chosen mesoscopic approach in which all dissipative phenomena are effectively lumped into a homogenised CZ model with an a priori assumed exponential traction-separation law. Figure 2a shows that this approach leads to spurious tractions at locations where the interface crack is fully developed (i.e., no material connectivity). Evidently, this does not reflect the actual physics.

To better understand the micro-mechanical dissipative mechanisms at the fibrillating delamination front, micron-scale experiments have been performed, thereby focusing on the fracture process zone.

## 4. Experimental Analyses at the Microscopic Scale

### 4.1. Visualization of Fibrillation

Figure 2b illustrates the occurring PDMS fibrillation during delamination, as visualized from the delamination front side of the sample. From a large number of ESEM images, it was observed that the fibrils initiate by delamination at the roughness peaks and remain attached to the valleys up to the moment of failure [28]. Initiated fibrils are elongated until they rupture or debond at the copper surface. Note that this mechanism is similar to that observed for the Thermoplastic Urethane-copper interface, as discussed in [27], where the same copper foil is applied. The initiation of fibrillation will be studied in more detail in Section 4.3 by means of an alternative peel test.

### 4.2. Material Behaviour of the PDMS Fibrils

#### 4.2.1. Deformation

**Figure 3:**
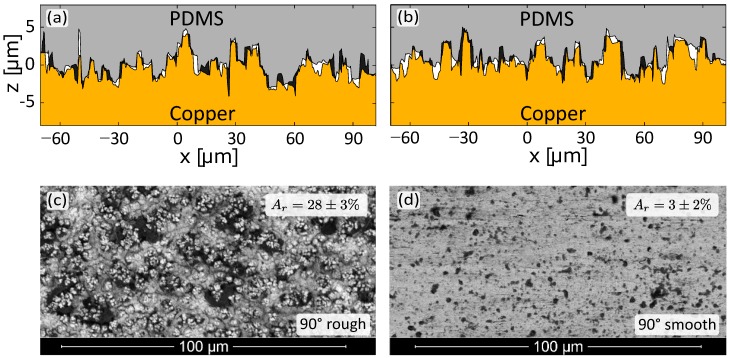
(**a**,**b**) reconstructed cross-sections at two locations from the obtained Copper-PDMS residual surface profile; (**c**,**d**) BSE images of the PDMS residue on the copper surfaces for the 90${}^{\circ }$ peel test; $A_{r}$ values are averaged over 10 images.

A probable dissipative mechanism during peeling that could enhance the macroscopic WOS, is non-elastic (i.e., dissipative) material behaviour around the delamination front. Previous studies showed that the dissipation from the elasto-plastic deformation in the copper foil is either negligible due to the small relative volume of the foil compared to the relatively large PDMS thickness [26], or it is significant but remains constant for other cases and does not contribute to the observed changes in WOS values [27]. To identify the occurrence of irreversible deformation during delamination in the PDMS material, the surface topologies of the separated fracture surfaces are compared. The 3D surface topology is measured with an optical profilometer (Sensofar Plμ 2300, Barcelona, Spain). The initial location of the PDMS surface with respect to the copper surface is recovered by applying a customized Global Digital Image Correlation (GDIC) routine, which is able to exploit the surface texture as a pattern (instead of applying a dedicated pattern) and match images of two distinct, separated surfaces (instead of two images of the same surface). For more details, the interested reader is referred to [28]. Due to the moulding process, the copper surface is initially fully covered by PDMS, as verified from a cross-sectional analysis [25]. Hence, the difference between the surface topologies indicates the level of irreversible deformation, under the fair assumption that algorithmic matching errors and contaminations are negligible. Examples of reconstructed cross-sections are depicted in Figure 3a,b. The mean amplitude of this difference is approximately 1 μm, which indicates that the average irreversible crack opening is smaller than 1 μm. For this reason, the dissipated energy is insignificant compared to the critical interface opening in the macroscopic CZ model (which is 74 μm for the smooth samples, and 148 μm for the rough samples). This confirms that the PDMS material primarily deforms in a hyper-elastic manner.

#### 4.2.2. Failure

In order to validate the occurrence of fibril rupture in the FPZ, the PDMS residue on the delaminated copper surfaces is analysed by using the Back Scatter Electron (BSE) composition contrast technique in the ESEM: a high contrast between the large atomic mass copper (bright) and the low atomic mass PDMS (dark). Figure 3c,d shows BSE detector images for each interface type and the 90${}^{\circ }$ peel test, with the average PDMS covered area $A_{r}$ indicated in the corner. The figures of the 0${}^{\circ }$ peel test are not shown here, as these exhibit similar results: for the rough surface, $A_{r}=33\pm 3\%$ while, for the smooth surface, $A_{r}=7\pm 2\%$. The fact that PDMS residue appears on the copper surface implies that the interface itself does not fail (which would not leave any PDMS residue) but instead the delamination propagates by fibril rupture. It can be observed that this fibril rupture mechanism is much more pronounced for the rough copper surface. Consequently, an increase in $A_{r}$ suggests an increase in the amount of energy dissipated during delamination, and, hence, a higher WOS [28].

### 4.3. Alternative Peel Test

The complex nature of the fibrillation process and its pronounced influence on the adhesion properties requires a more comprehensive understanding of the relevant mechanisms at the micro-scale, deeper within the FPZ. In the results discussed so far, based on References [25,26,27,28], fibrillation could not be observed directly from the side face of the peel front, due to a lack of resolution from the optical microscope and because of edge effects masking the field of view. Therefore, an alternative peel test is introduced in this contribution, which allows for observation of a pristine fibrillation front from the side with sufficient resolution.

**Figure 4:**
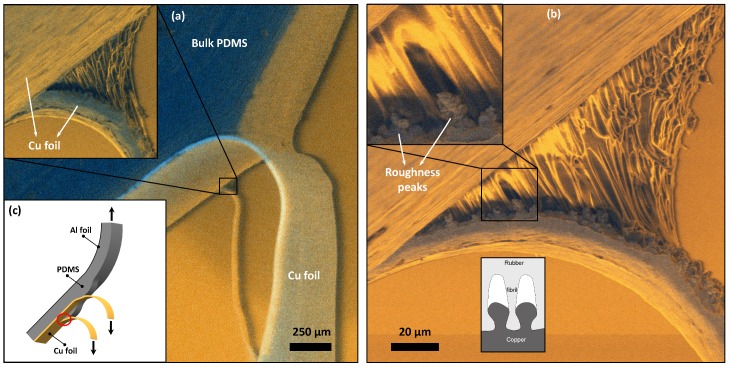
The alternative peel test: (**a**) two active peel fronts occur with the inset showing a detailed side view of the fibrillating PDMS; (**b**) close-up of the fibrillation front clearly indicating, for the first time, that initiation occurs at the roughness peaks while the PDMS is strongly anchored in the roughness valleys; (**c**) schematic of the alternative peel test.

This peel test is conducted using the same rough copper samples from Section 2 in a 180${}^{\circ }$ remote loading configuration (see Figure 4c). An aluminium foil is glued to the backside of the PDMS to avoid large bulk deformation while the copper foil is cut in two parts along the length direction of the sample, up to the initial peel front. The loading state and geometry of the alternative peel test is shown in Figure 4c. The peel test is prepared by inserting the PDMS and aluminium foil in one clamp of the tensile stage and the two parallel copper flaps on the other side, where one flap is fixed approximately 750 μm further in the clamp than the other. After subsequent 90${}^{\circ }$ rotation of the clamps to facilitate the side view, the peel test is started at a rate of 0.1 μm/s in the ESEM. Upon reaching steady state peeling, Secondary Electron (SE) and BSE imaging is combined, after elaborate optimisation for a minimal electron invasive approach, to capture the details of the fibrillation deeper in the peel front.

The special copper clamping approach yields two separate peel fronts, one more advanced than the other, while the two copper flaps continuously tear and expose a pristine side view of fibrillation, as is illustrated in Figure 4a. The size of the FPZ near the tearing copper is significantly smaller compared to the regular peel front geometry because the other (non-peeling) copper flap prevents lift-off of the bulk PDMS. This indicates that the force-displacement behaviour will be different due to the tearing contribution. However, the behaviour of the fibrillation process and the fibril length is similar to what is observed in Figure 2b, which confirms that this particular experiment can indeed be used to study the initiation of fibrillation deeper in the FPZ.

Figure 4b depicts a close-up of the side of the fibrillating front. Individual fibrils can be distinguished deep into the FPZ. This point of view yields a remarkably clear visualisation of the copper roughness peaks. Moreover, initiation of the fibrils is identified as a result of cavitation on top of the roughness peaks. This behaviour can be explained by large hydrostatic stresses at the peaks that initiate cavitation, followed by fibrillation [44], as schematically shown by the inset of Figure 4b.

Consequently, the experimental analyses at microscopic scale provide the following conclusions: (1) PDMS exhibits hyper-elastic, non-dissipative, behaviour; (2) fibril rupture occurs in the FPZ; and (3) the fibrils are initiated at the peaks of the copper roughness profiles. These characteristics are now incorporated into single and multiple fibril models at the microscopic scale to enable quantitative prediction of the macroscopic WOS for the Cu-PDMS system.

## 5. Numerical Analyses at the Microscopic Scale

### 5.1. The Single Fibril Model

**Figure 5:**
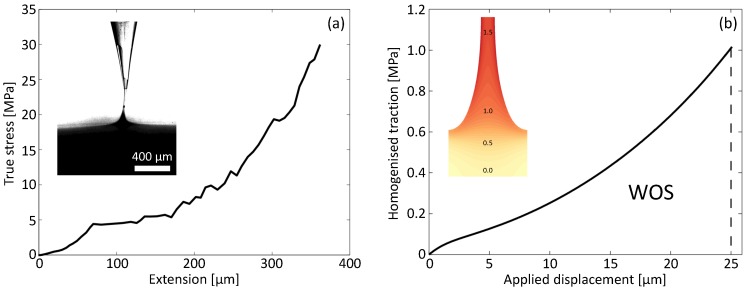
(**a**) the PDMS single fibril experiment: example of a measured maximum true stress–extension curve during stretching, the inset shows drawing of the material from the hollow tip into the fibril, while simultaneously stretching the fibril, just before fibril rupture; (**b**) homogenised traction–separation response obtained from the single fibril micro-model, the inset shows the final stage of the simulated fibrillation process in which colours indicate the maximum principal true strain as indicated by the numbers.

First, the contribution of fibril mechanics to the WOS is quantified by means of a micromechanical model of a single fibril, in which the growth of a nucleated fibril up to the moment of fracture is described. Given the large variation in measured stress-strain curves for rubber materials reported in the literature [55], a small scale single fibril experiment is performed to characterize the material properties. In the experiment, hollow tips are fabricated from glass tubes (outer diameter 1 mm, inner diameter 0.58 mm). The tip is attached to an aluminium cantilever having a stiffness of 13 N/m. After mixing and degassing, the PDMS is poured into an aluminium container. The container is moved towards the tip in order to form a capillary bridge by means of a micro-precision stage. The PDMS is cured for 1 hour at 100${}^{\circ }$ by a heating stage. After cooling down to room temperature, the load is applied at a rate of 30 μm/s. The experiment is visualized by a Zeiss V20 optical microscope (Oberkochen, Germany) coupled to a Zeis Axiocam HR camera, which records three frames per second. The tip is tracked using GDIC [56], which is optimized to deal with sparse patterns and rigid body displacements. The resulting fibril force is accurately measured from the movement of the tip by using the known (low) cantilever stiffness. The maximum true stress in the fibril is calculated in the smallest cross-section, which is obtained from tracking the contour of the fibril using a gray value thresholding method. The resulting fibril fracture stress values are extracted from three successful experiments, and range between 25 and 30 MPa, showing a relatively low variability [55]. An example of a resulting stress–extension curve is shown in Figure 5a in which the inset shows the single fibril experiment just before fibril rupture. Note that the parameter identification procedure was performed on the PDMS fibril outside the glass tip, thereby preventing any influence of the glass–PDMS interaction.

To capture the strain hardening behaviour in the fibrils due to the locally occurring large strains, the mechanical behaviour of the PDMS material is described by a more sophisticated hyper-elastic Ogden material model. The particular form of the strain energy density function is taken from [57]

(5) $$W=\frac{1}{2}\kappa {\left(J-1\right)}^{2}+\sum_{k=1}^{N}\frac{c_{k}}{m_{k}^{2}}\left(\lambda_{1}^{m_{k}}+\lambda_{2}^{m_{k}}+\lambda_{3}^{m_{k}}-3-m_{k}\ln J\right)$$

with J=det(F) the volume change ratio, F the deformation gradient tensor, $c_{k}$ and $m_{k}$ are material parameters, and κ is a penalty parameter to enforce the near incompressibility of the PDMS material. The constitutive parameters are provided in Vossen et al. [55]. Fibril failure is described by means of a critical stress criterion, extracted from the single fibril experiment, and is defined as the average true stress in the smallest cross-section of the fibril.

An example of the simulation of the growth of a fibril from an initially stress-free state up to the moment that the true stress reaches the critical value is depicted by the inset in Figure 5b, which clearly illustrates the large non-uniform deformations within the fibril, which was simulated by employing a large deformation adaptive FE discretisation strategy [58]. The macroscopic interface properties are established from the single fibril micro-model by means of a multi-scale interface model, based on computational homogenisation [58,59,60,61,62]. Here, the macroscopic traction vector $T_{M}$ is determined for each macroscopic opening vector $u_{m}$, which corresponds to the displacement applied to the micro-model. For the prescribed mode I loading, the macroscopic first Piola–Kirchhoff traction vector $T_{M}$ can be determined from the Hill energy condition. In fact, for the considered micro-model boundary conditions, it is simply the average traction at the top of the single fibril model, $T_{M}$. From the micro-model simulations, the macroscopic traction-separation response (TSR) is obtained (see Figure 5b). The typical strain hardening behaviour at large deformations is prominent. Note that the frequently used exponentially decaying traction-separation response is not recovered; instead, the traction abruptly drops to zero at the moment of fibril failure. The importance of the shape of the TSR will be emphasized in more detail in Section 6. From the TSR, the macroscopic WOS is determined

(6) $$\Gamma_{i}=\int_{0}^{u_{m}^{\max}}T_{M}\;du_{m}$$

To reflect the variability in fibril properties observed in the peel tests, a range of values for the initial fibril dimensions is used in the simulations. The maximum WOS value from the single fibril simulations is $\Gamma_{i}=18.2$ J$m^{-2}$, while the homogenised strength is approximately 1.0 MPa (see Figure 5b). Even though this WOS value is significantly larger than the typical values for the micro-scale adhesion energy, it is still an order of magnitude smaller than the values obtained from the peel tests in Section 3. It can thus be concluded that the single fibril micro-mechanics model does not contain all relevant dissipative mechanisms at the micro-scale to explain the high WOS values. For this reason, a discrete multi-fibril model has been developed to analyse the interaction between the fibrils as well as the interaction between fibrils and the adjacent bulk rubber.

### 5.2. The Multiple Fibril Model

To analyse mixed-mode sequential failure of the fibrils, a multiple fibril model was developed. Without compromising the physics of the underlying dissipative processes, the failure of the fibrillar structure is approximated by assuming that fibrils fail instantaneously once the magnitude of the average first Piola–Kirchhoff stress *p* acting on the fibril’s top end reaches the critical stress $p_{c}$ [55]. The failed fibrils are unable to transfer any load and are further discarded during the simulation. The interaction between substrate and fibril is captured through appropriate boundary conditions that are directly applied to the fibrils, allowing free contraction and rotation of the fibril top end. In [63], it was shown that the type of boundary conditions applied at the fibril top end has a minor effect on the resulting response under mode I loading. Moreover, since the focus is on the effect of the discrete fibrils on the bulk behaviour, whereby boundary conditions are applied at the fibril top end, their influence on the final results is expected to be negligible.

The importance of the discreteness of the fibrils was illustrated by calculating the WOS as function of fibril width in the case of simultaneous fibril failure under mode I loading [64]. This simplified loading case has been often studied in literature on fibrillar structures [33]. The obtained results suggested that the absolute value of the spacing between the fibrils significantly affects the energy loss in the bulk. However, for sufficiently separated fibrils, the energy loss in the bulk due to the fibrils increases significantly. It is therefore essential to properly account for the discrete character of the system when studying delamination mechanisms in fibrillating interfaces.

**Figure 6:**
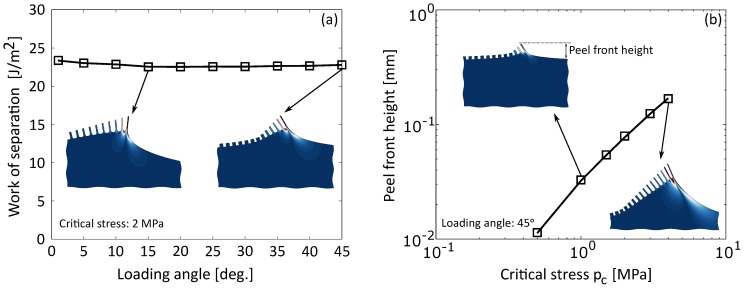
(**a**) WOS as function of loading angle, with the insets showing deformed geometries for two loading angles 15${}^{\circ }$ and 45${}^{\circ }$ at $p_{c}=2.0$ MPa (the colours indicate the strain energy density (SED, (N/mm${}^{2}$)) after failure minus the SED before failure, projected on the geometry before failure). The WOS is not significantly influenced by the loading angle; (**b**) peel front height as function of critical stress, with the insets showing deformed geometries for two critical stress values $p_{c}=1.0$ MPa and $p_{c}=4.0$ MPa at a loading angle of 45${}^{\circ }$. The critical stress has a significant influence on the peel front height and the WOS value.

Sequential fibril failure was considered while varying the loading angle in the simulations. The insets in Figure 6a show the calculated deformed geometries for two loading angles at $p_{c}=2$ MPa. It can be observed that for all loading angles that a volume of bulk material is highly deformed near the critical fibril. Figure 6a shows that the resulting WOS is not significantly influenced by the loading angle, which may seem surprising, as mode dependency is a commonly reported phenomenon for interface fracture toughness values [65,66]. However, as discussed in Section 3, peel tests on these particular metal–elastomer fibrillating interfaces reveal a negligible mode dependency. These numerical results therefore provide a physical explanation for these experimental observations: as the load transmitted by the fibrils on the bulk is nearly equal for all loading angles, the amount of energy stored and subsequently released in an unstable manner in the bulk is not considerably changed by the specific loading angle [64].

Next, the effect of the critical stress is studied. For this purpose, the critical stress value is varied between $p_{c}=0.5$ MPa and $p_{c}=4.0$ MPa. These values are motivated by the calculated homogenised single fibril strength (Figure 5b) and assuming a surface area fraction of 50% between fibrils and crack surface. Examples of the resulting deformed geometries are shown in the insets of Figure 6b, at a loading angle of 45${}^{\circ }$. Comparison of the calculated peel front geometries with the experimentally observed lift-off geometry (e.g., Figure 1) confirm the pertinence of the applied loading conditions. The calculated peel front height (defined in the left inset), and hence the size of the fracture process zone, as function of the critical stress is shown in Figure 6b, and suggests a correlation between the WOS value and the peel front height.

Figure 8 depicts the WOS as a function of the peel front height from the simulation results, for all considered fibril widths [64]. The pronounced effect of the critical stress, and thus the peel front height, on the WOS can be clearly recognised. Earlier reported experimentally measured values [25,26,28] are included in the figure as black circles, where the difference between the two ‘rough substrate’ data points (b) and (c) is caused by the difference in PDMS thickness used (0.75 mm versus 1.0 mm), which emphasizes the importance of bulk PDMS activation in the total fracture toughness. The predicted WOS values from the numerically obtained peel front height are in good agreement with the experimentally measured values. This suggests that the high WOS values can be explained by the following phenomena: (i) the dynamic release of the stored elastic energy by fibril fracture; (ii) the spatial discreteness of multiple fibrils as well as the interaction of these fibrils with the adjacent deforming bulk elastomer material; and (iii) the highly nonlinear behaviour of the elastomer. Details of this analysis can be found in [64].

## 6. Macro-Scale CZ Model Revisited: A Fibril-Motivated TSL

As discussed in Section 3, and in particular in Figure 2a, the commonly used exponential TSL in CZ models does not adequately describe the delamination behaviour of the PDMS-Cu samples. In fact, the detailed multi-scale analyses (Section 4 and Section 5) suggest an alternative TSL shape: an exponentially increasing traction up to $t_{\max}$, followed by a sudden traction drop to zero (i.e., an infinitely large negative tangent stiffness), as seen in Figure 5b. The sudden drop results from the fact that this response is characteristic for a single fibril. Clearly, a delamination front with numerous failing fibrils with a statistical variation in strength values will result in a more gradual drop (i.e., a finite negative tangent stiffness) after reaching the homogenised strength $t_{\max}$. Indeed, in literature, it is suggested that the TSL affects the delamination behaviour [67,68,69]. In fact, Li and Chandra [70] argue that the shape is dependent on the microstructural dissipative mechanisms. An additional result of the multi-scale analyses is the established quantitative relation between the WOS and the peel front height.

**Figure 7:**
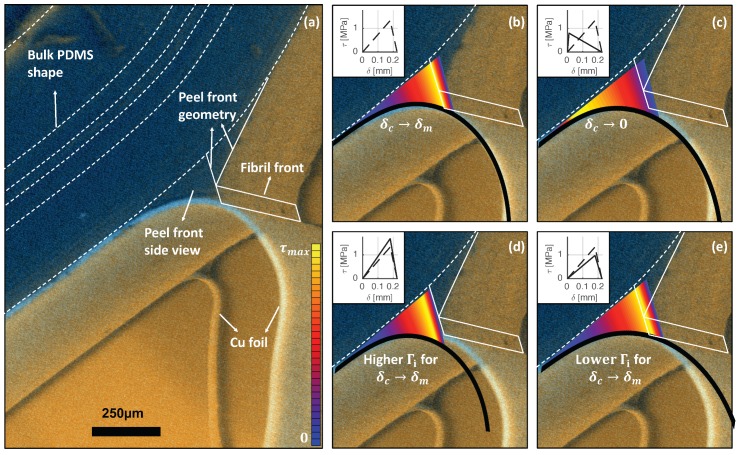
Side view of the ESEM images of the alternative 180${}^{\circ }$ peel test to which the simulations are matched. (**a**) the shape of the bulk PDMS is marked with white dashed lines, recognised under high contrast in the PDMS. The peel front geometry is drawn with solid white lines, indicating the peel front side view and the fibril front; (**b**) the best-matching simulation (corrected for the angle of observation) superposed on the experimental image, with the applied bilinear TSL in the inset. The copper foil from the simulation is depicted in black, and the acting tractions in the CZ are colour coded. An adequate agreement with the simulation is observed for the copper curvature and the FPZ shape; (**c**–**e**) overlays of simulations that do not accurately represent the copper curvature, with each used TSL depicted as a solid line in the inset, while the best-matching TSL corresponds to the dashed line. These simulations clearly show the sensitivity of the CZ parameters and shape with respect to the copper curvature and the peel front shape.

In this section, these main findings will be assessed by means of the alternative 180${}^{\circ }$ peel test, introduced in Section 4.3. This test results in two side-views of separate process zones: the small fibrillating front exposed by the copper tearing and the actual side of the sample where ‘regular’ PDMS lift-off can be observed without any direct evidence of fibrils. While the small peel front results in a detailed view on the fibrillation deep in the process zone, as shown in Figure 4, its smaller size is caused by the additional constraint of the nearby copper, which complicates comparison with simulations. However, the regular 180${}^{\circ }$ peel front on the actual side of the sample can still be exploited to characterise the macroscopic adhesion properties. Figure 7a depicts the bulk PDMS shape, the geometry of the peel and fibril front and copper curvature.

### The Pronounced Effect of the TSL Shape

Similar to Section 3, an FE model with CZ elements is used to characterise the adhesive properties of this alternative loading state and to get a better understanding on the effect of the TSL law, in terms of its shape and properties. The elasto-plastic copper foil of 17 μm is attached to the CZ elements and the simulation is started by pulling the free end of the copper foil down, according to the experimental configuration in Figure 4c. After steady state peeling is reached, the peel front shape is captured and compared to the experimental observations, accounting for the angle of observation. To estimate the impact of a different TSL shape compared to the conventional exponential TSL, a bilinear TSL is used (e.g., [71]) in order to approximate the TSL depicted in Figure 5b:

(7) $$t\left(\delta \right)=\left\{\begin{array}{cc} \frac{t_{\max}}{\delta_{c}}\delta \; & \mathrm{for}\;0\leq \delta \leq \delta_{c}\\ \frac{t_{\max}}{\delta_{m}-\delta_{c}}\left(\delta_{m}-\delta \right) & \mathrm{for}\;\delta_{c}<\delta \leq \delta_{m}\\ 0 & \mathrm{for}\;\delta >\delta_{m} \end{array}\right.$$

Here, $\delta_{c}$ is the opening at $t=t_{\max}$, while at opening $\delta_{m}$ the traction becomes zero. For this bilinear TSL, the interface toughness reads

(8) $$\Gamma_{i}=\int_{0}^{\infty }t\left(\delta \right)d\delta =\frac{1}{2}t_{\max}\delta_{m}$$

The values of the CZ parameters are obtained by minimizing the difference between the shape of the deformed PDMS and the curved copper foil at the delamination front as determined from the simulations and the experiments, similar to [25,26]. Examples of the comparison of several TSLs with the experiment are given in Figure 7b–e, in which the thick black line indicates the copper foil and the CZ elements are coloured by their traction value. For each TSL, de traction-separation curve is given in the insets. The parameter set that best describes the delamination front consists of $\Gamma_{i}=150$ J$m^{-2}$, $t_{\max}=1.33$ MPa, $\delta_{c}=0.18$ mm and $\delta_{m}=0.22$ mm, of which the result is shown in Figure 7b. Notice that the thus identified $t_{\max}$ value is similar to the homogenised single fibril value of 1.0 MPa (see Figure 5b).

To assess the significance of the actual value of the negative tangent in the TSL (i.e., the part of the TSL between $\delta_{c}$ and $\delta_{m}$), two limiting values for $\delta_{c}$ are compared: (1) $\delta_{c}\to \delta_{m}$ (Figure 7b) and (2) $\delta_{c}\to 0$ (Figure 7c), where it is noted that for case (2) the remaining CZ parameters are optimized to give the best correspondence. It can be observed that the curvature profile along the FPZ and thus the shape of both the PDMS and the copper foil are affected by this value: lower $\delta_{c}$-values result in larger deviation of these shapes compared to the experiment, which corroborates the earlier found high value of the negative tangent. The effect of a variation of 25% in $\Gamma_{i}$, with constant values for $\delta_{c}$ and $\delta_{m}$, is illustrated in Figure 7d,e: the entire curvature profile of the copper foil is affected by $\Gamma_{i}$, while the size of the FPZ does not significantly alter. It can thus be concluded that $\Gamma_{i}$ affects the entire shape of the copper foil, whereas $\delta_{c}$ influences both the shape of the copper foil and the FPZ, locally at the fibrillation front. Hence, both parameters can be determined uniquely. Detailed analysis of the small remaining identification residual in Figure 7b suggests that a nearly perfect match can only be obtained with an exponentially increasing TSL up to $\delta_{c}$, yielding an optimal TSL that is quite similar to the single fibril traction-separation response, shown in Figure 5b. These results clearly show that, besides the TSL parameters, the TSL shape is also important in adequately describing delamination in stretchable electronics applications.

**Figure 8:**
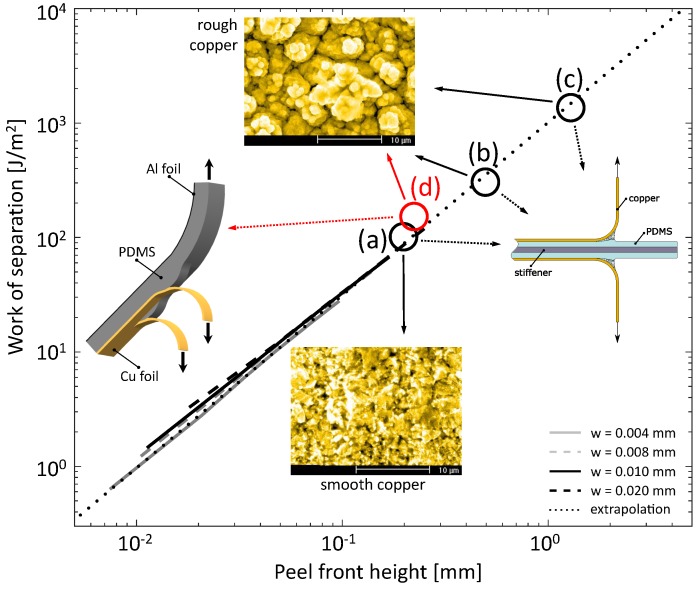
Work-of-separation versus the peel front height including the result of the new experiment indicated by the red circle. Earlier reported experimentally measured values from the 90${}^{\circ }$ peel test are included in the figure as black circles: (**a**) smooth copper, $h_{\mathrm{PDMS}}=0.75$ mm [28]; (**b**) rough copper, $h_{\mathrm{PDMS}}=0.75$ mm [28]; (**c**) rough copper, $h_{\mathrm{PDMS}}=1.0$ mm [25,26]; (**d**) alternative 180${}^{\circ }$ peel test result.

As additional validation, these newly obtained values for $\Gamma_{i}$ and peel front height are included in the WOS-peel front height curve as a red circle (see Figure 8). The samples are significantly different in geometry and loading condition, which is reflected by an order of magnitude difference in the peel front height for the three peel tests performed on the same rough copper specimen (data points (b), (c), and (d) in the figure). Nevertheless, the new data point shows an adequate agreement with the earlier established relation between the WOS and the peel front height. This proves that the relevant mechanisms are equal for the different samples and, therefore, it is concluded that the key parameter that controls the WOS is the peel front height. The actual sample design parameters such as PDMS thickness and backside layer(s) are only of secondary influence through the peel front height.

## 7. Discussion

A multi-scale experimental and numerical analysis, encompassing a range of analysis techniques, has been employed to unravel the physical origin behind the high macroscopic WOS of large elastic mismatch interfaces, particularly the Cu-PDMS system, as a function of the interface roughness. For this purpose, previously published results were collected and retrospectively analysed. The remaining open questions were addressed by providing new and independent results obtained from an alternative experimental set-up. The experimental investigation, including 0${}^{\circ }$, 90${}^{\circ }$, and 180${}^{\circ }$ peel tests, revealed that the delamination mechanics is a multi-scale problem spanning all length scales. The soft elastomer exhibits large deformations in the vicinity of the peel front, and microscopic images of the lift-off geometries were successfully applied to characterize the properties of a CZ model, i.e., the interface toughness, critical opening, and mode angle dependence of an exponential TSL. Analyses of the FE simulations revealed two intriguing observations: (i) the presence of non-physical tractions in regions where no material is present; and (ii) a negligible mode angle dependency in contrast to reports in the literature.

These observations suggest a complex delamination process. Therefore, the progressing peel front was imaged in situ in an Environmental Scanning Electron Microscope (ESEM). Front-view visualization at high magnification revealed that, at the peel front, a fibrillation process occurs, while high-magnification side-view visualization clearly showed, for the first time, that the fibrils initiate at the peaks of the copper roughness profiles. The fibril shape, distribution (and hence, discreteness), and location were found to be governed by the copper topography, which was explained by a mechanism of fibril nucleation resulting from a combination of mechanical interlocking at roughness valleys and cavitation at the roughness peaks. In other words, an inhomogeneous micro-scale interface load triggers the initiation of the fibrillation process, which enhances the macroscopic WOS. These results suggest that fracture toughness improvement must be feasible by engineering particular surface topographies.

Quantification of the PDMS residue on delaminated copper surface revealed that the delamination propagates primarily by fibril rupture instead of interface decohesion, while quantitative matching of the two crack surface topologies showed that the PDMS material deforms in a fully hyper-elastic manner. With these microscopic observations at hand, a single fibril model was developed, which was calibrated to dedicated PDMS single fibril experiments. The single fibril simulations showed that, contrary to the frequently used exponentially decaying TSL, the fibril exhibits a nonlinear increase in traction with increasing opening displacement up to the point of a sudden loss of traction due to fibril fracture. Detailed analysis of different TSL shapes to a side view of the microscopic peel front confirmed that a TSL that describes abrupt failure provides the most accurate simulation of the fibrillation process, which underlines the key role of the TSL shape for accurate delamination predictions in these stretchable electronics interface systems.

Finally, a discrete multiple fibril model that incorporates this abrupt fibril rupture was employed to analyse the mutual interaction between the fibrils as well as the local transfer of loads to the adjacent bulk rubber. The amount of energy stored and released in an unstable dynamic way is not significantly affected by the loading angle, which is supported by the experimental findings. Furthermore, the high WOS values were explained by the unstable dynamic release of stored elastic energy in the PDMS bulk, the spatial discreteness of the fibrils, including the interaction of the fibrils with the adjacent deforming bulk materials, and the highly nonlinear behaviour of the PDMS at large strains. The results established an experimentally validated quantitative relation between the peel front height and the macroscopic WOS, which was confirmed by the new and independent results from the alternative peel test.

The rigorous multi-scale approach presented here, combining experimental and numerical analysis to fully identify and quantify the dissipative processes involved in the delamination process, can be applied to other material systems frequently encountered in plane stretchable electronics designs, such as polymer/metal/polymer stacks and metallic nanofilm-PDMS systems e.g., [3,10], even though the specific relevant dissipative mechanisms might be different. It is expected that, for metal–elastomer interface systems, fibrillation occurs in the case of sufficient intrinsic adhesion combined with pronounced surface roughness-induced mechanical interlocking, with the latter defining the fibril spatial distribution.

From these considerations, one seemingly straightforward way of increasing the WOS, is to enhance the strength of the PDMS by increasing its cross-link density, as this will delay fibril rupture and thus increase the dissipated, elastically stored energy in the PDMS bulk. However, the multi-scale observations suggest the existence of a delicate balance between the fibril debonding at the interface and fibril rupture. If the (micro-scale) fibril-copper adhesion is insufficient, the fibrils will debond before the full dissipative capacity of the PDMS bulk is reached. To prevent this, a strongly interlocking structure is required at the base of the fibrils. One way to achieve this is by employing similar high roughness interfaces as the ones investigated here, which are indeed being applied in current large-area stretchable electronics (demonstrators). For microelectronics applications with (much) smaller interconnect sizes, it is interesting to see if an artificial roughness, specifically designed to initiate cavitation and interlocking, can initiate fibrillation, control fibril discreteness, and provide similar enhancements in fracture toughness at the micro-scale. A dedicated micro-mechanical model that quantitatively predicts interlocking effects on the macroscopic WOS by means of describing the competition between cohesive and adhesive failure (similar to e.g., [72,73,74,75]) could be developed to optimize the surface pattern at microscopic scale for tailored WOS values. In this way, (lithography-)controlled height patterns or small-scale, fractal-like patterns could be studied while taking into consideration the influence of possible edge effects.

## Abbreviations

The following abbreviations are used in this manuscript:

BSE	Back Scatter Electron
Cu	Copper
CZ	Cohesive Zone
DIC	Digital Image Correlation
ESEM	Environmental Scanning Electron Microscopy
FE	Finite Element
FPZ	Fracture Process Zone
GDIC	Global Digital Image Correlation
PDMS	Poly(dimethylsiloxane)
PSA	Pressure-sensitive adhesive
SE	Secundary Electron
SED	Strain energy density
TSL	Traction Separation Law
WOS	Work-of-separation
